# The Relationship Between Clinical Leadership Behaviors, Flourishing, and Extra-Role Behavior Among Nurses: A Cross-Sectional Study

**DOI:** 10.1155/jonm/5438358

**Published:** 2025-04-21

**Authors:** Wedad Khader, Ghada Abu Shosha, Islam Ali Oweidat, Saleh Al Omar, Mohammad R. Alosta, Abdulqadir J. Nashwan

**Affiliations:** ^1^Faculty of Nursing, Zarqa University, P.O. Box 13132, Zarqa, Jordan; ^2^Maternal and Child Health Nursing Department, Zarqa University, P.O. Box 13132, Zarqa, Jordan; ^3^Community and Mental Health Nursing Department, Faculty of Nursing, Zarqa University, P.O. Box 13132, Zarqa, Jordan; ^4^Faculty of Nursing, Al-Balqa Applied University, P.O. Box 19117, Salt, Jordan; ^5^Clinical Nursing Department, Faculty of Nursing, Zarqa University, P.O. Box 13132, Zarqa, Jordan; ^6^Department of Nursing, Hamad Medical Corporation, P.O. Box 3050, Doha, Qatar

**Keywords:** clinical leadership, extra-role behavior, flourishing, leadership and well-being, leadership-behavior relationship

## Abstract

**Background:** Clinical leadership is vital in improving nurses' performance and healthcare services.

**Aim:** This study examined the relationship between clinical leadership behaviors, flourishing, and extra-role behavior among nurses in Jordan.

**Methods:** A descriptive correlational design was used, which enrolled a convenience sample of 260 registered nurses working at governmental hospitals in Jordan.

**Results:** The mean score of clinical leadership behaviors was moderate (*M* = 138.01, SD = 7.82). Nurse flourishing had a mean total score of (*M* = 31.76, SD = 5.49), indicating positive well-being. Extra-role behavior had a mean score of (*M* = 23.96, SD = 3.93), indicating moderate participation in extra-role behaviors. There was a significant moderate positive correlation between clinical leadership behaviors and nurse flourishing (*r* = 0.489, *p*=0.047) and extra-role behaviors (*r* = 0.359, *p*=0.004). Factors that significantly predicted nurse flourishing and extra-role behavior were clinical leadership behaviors, age, duration of nursing experience, income, and hospital number of beds.

**Conclusion:** Effective clinical leadership behaviors can significantly improve nurses' flourishing and extra-role behavior.

**Implication for Nursing Management:** The results emphasize the importance of training nurses on positive leadership behaviors to enhance flourishing and involvement in extra-role behavior.

## 1. Introduction

Nurses have a vital role in healthcare systems. They provide comfort and well-being to patients [1], which require commitment, empathy, and professionalism [2]. Beyond their formal role, nurses have many relationships with patients, families, coworkers, and others, which encourage them to perform other tasks not mentioned in their job description, such as taking part in quality improvement activities and patients' rights activities, among others, and creating a healthy workplace [1, 3]. This extra-role behavior (ERB) could positively impact nurses' satisfaction and morale [1]. Nurse flourishing is defined as a state of holistic well-being that incorporates several dimensions of positive mental and psychological health. It is characterized by positive emotions, participation, interest in daily activities, and optimism [4]. Nurse flourishing is important in increasing nurses' satisfaction, retention, and quality of healthcare [1, 5].

Clinical Leadership (CL) refers to all activities, practices, and traits expressed by nurses to lead, influence, and inspire their colleagues, consequently promoting their performance and enhancing the quality of patient care [2]. It was documented that a supportive leadership style has a positive impact on nurse job satisfaction [6], work relationships, the overall efficiency of healthcare systems [2], organizational commitment [6, 7], coping among the nursing workforce [8], engagement, the feeling of value, and motivation among nurses in Jordan [9]. On the other hand, poor or weak leadership may result in higher burnout rates and a lower level of nurse involvement [10], slowing down various organizational innovations, cooperation, and performance enhancement efforts [8].

Leadership style can also improve ERB, which refers to voluntary actions performed by nurses that go beyond their formal job responsibilities [3]. Examples include mentoring new staff, participating in quality improvement initiatives, advocating for patient rights [3], peer coaching [1], assisting coworkers, going the extra mile to search for solutions, and lobbying for change within organizations to improve workplace climate and patient outcomes [11]. ERB is not only beneficial for the work environment but also for team cohesiveness and organizational effectiveness [12].

The theoretical framework underpinning this study links these three concepts: CL, nurse flourishing, and ERB. Understanding the relationship between CL, nurse flourishing, and ERB is vital to understand how nursing leadership can affect nursing practice and the quality of healthcare. This could guide the development of interventions and policies tailored to the unique challenges faced by nurses [6]. Despite the recognized importance of CL, prior research has mainly addressed Western healthcare systems, creating a substantial gap in understanding how these CL behaviors (CLB) work in non-Western countries, predominantly in Middle Eastern settings like Jordan, where nurses comprise a large percentage of healthcare workers. Hence, little is known about how the cultural, organizational, and contextual factors potentially influencing the association between CLB and nurse outcomes in Jordan [2]. Filling this gap could help explain the role of leadership and favorable working conditions in encouraging nurses to go beyond their formal job requirements, thereby advancing nursing practice, improving organizational performance, and strengthening the quality of healthcare services. Therefore, this study aimed to investigate the relationship between CLB, flourishing, and ERB among nurses working at governmental hospitals in Jordan. A secondary aim was to identify the predictors of flourishing and ERB. The research questions guiding the study were; what are the relationships between clinical leadership behaviors and nurse flourishing, and between clinical leadership behaviors and ERB among Jordanian nurses working in governmental hospitals? and what are the predictors of nurse flourishing and ERB among Jordanian nurses working in governmental hospitals?

## 2. Methods

### 2.1. Study Design

This research study used a descriptive correlational cross-sectional design. This approach allowed the investigation of multiple variables simultaneously and provided insight into the antecedents of flourishing and ERB. Furthermore, it aids in collecting data from a large number of participants [13]. It also investigated relevant research problems [14–16].

### 2.2. Study Participants

The target population is registered nurses working in governmental hospitals in Jordan. However, only nurses from three large governmental hospitals were accessible. The tertiary hospitals provide wide healthcare services, including emergency, medical, surgical, and outpatient care. Two are in the country's capital, where around half its population lives. The first is a medical center with 1256 beds divided into four smaller hospitals. The second hospital is a teaching hospital that has 400 beds. The third hospital is located in Jordan's second-largest city, with a capacity of 300 beds [17].

Nurses who were registered nurses, had at least 1 year of experience, and could read and write in English were eligible to participate in the study. Nurses working in administrative positions, having formal leadership and administration training, or not holding a baccalaureate degree was excluded.

A convenient sample of nurses was recruited from the three hospitals. The sample size was calculated using the G^∗^Power software version 3.1 [18]. It was calculated for the linear multiple regression using eleven possible predictors and the following parameters: (power = 0.8), (Alpha = 0.05), (effect size = 0.07), and two-tailed. The estimated sample size was 251 participants. However, it was increased by 15% to compensate for possible withdrawal from the study and incomplete data (*N* = 289) [19].

### 2.3. Study Tools

The researchers developed a questionnaire to assess different characteristics, including sociodemographic (e.g., age, gender, marital status, and educational level), work-related (e.g., duration of nursing experience, specialty area, and monthly income), and organizational (e.g., hospital size and nurse-patient ratio).

The CLB Questionnaire (CLB-Q) assessed nurses' perceptions of their CLB. It is a self-reported questionnaire consisting of 46 items rated on a 5-point Likert scale with scores ranging from (1) “never” to (5) “always.” It encompasses seven subscales: self-awareness (6 items), decision-making (7 items), advocacy and empowerment (6 items), communication (6 items), teamwork (6 items), quality and safety (7 items), and clinical excellence (8 items). The total score could range from 46 to 230 [20]. When converted to percentages, scores could be categorized into three levels: 0%–33.3% indicating low participation in CLB, 33.4%–66.6% indicating moderate participation in CLB, and 66.7%–100% indicating high participation in CLB [20]. The internal consistency Cronbach's alpha coefficients of the instrument subscales ranged from 0.88 to 0.94 [20] and 0.96 for the whole instrument [6].

This Flourishing Scale assessed nurses' perceptions of flourishing, encompassing participation, interest in daily activities, purpose, and optimism. It is a self-report questionnaire composed of eight items rated on a seven-point Likert scale, with scores ranging from (1) “strongly disagree” to (7) “strongly agree.” The total flourishing score can range from 8 to 56. This instrument had strong psychometric properties [21, 22], including high internal consistency with a Cronbach's alpha coefficient of 0.87 [21] and good convergent validity [23].

The ERB Scale assessed nurses' perceptions of their participation in ERB. It is a self-report questionnaire consisting of eight items rated on a five-point Likert scale, ranging from (1) “never” to (5) “always.” The total score ranges from eight to 40. Cronbach's alpha coefficient of internal consistency was 0.94 [24].

### 2.4. Study Processes

Ethical approvals were obtained from the Institutional Review Board (IRB) at the Faculty of Nursing at Zarqa University No. 15/2023 and the targeted hospitals. Additional approval to conduct the study was also obtained from the administration departments of the three hospitals. Then, the researchers visited each hospital to introduce the study to nursing managers and hand over invitation letters outlining the study aims, methods, and confidentiality measures. After that, potential participants were identified with the help of nursing managers. Then, nurses were approached by the researchers, who explained the study to them and asked nurses willing to participate to sign informed consent to ensure their voluntary participation.

Data collection extended between May and July 2024. A paper-based questionnaire was administered in English and took approximately 15–20 min to complete. A quiet room was used to enable nurses to fill out the questionnaire. Then, they handled the filled responses to the researchers by the next day. The filled responses were securely stored in a locked cabinet based on the institutional guidelines for data retention. In addition, participants' confidentiality and anonymity were maintained during the study.

### 2.5. Data Management

Statistical Package for the Social Sciences (SPSS) Statistics version 26 software was used to analyze the data. Missing data were replaced using multiple imputation techniques to ensure incomplete responses did not bias the analysis. All assumptions of the statistical tests, including normality, linearity, homoscedasticity, and independence of residuals, were thoroughly checked and met. Moreover, descriptive statistics (frequencies, percentages, means, and standard deviation) were used to describe nurses' perceptions of CLB, flourishing, and ERB. Pearson correlation test was used to examine the relationship between CLBs and nurses flourishing and ERB. Multiple regression analysis was also used to analyze the predictors of nurse flourishing and ERB. The level of significance was set at (*p* < 0.05).

## 3. Results

### 3.1. Sociodemographic and Work-Related Characteristics of the Nurses

The researchers approached 289 nurses. However, a total number of 274 agreed to participate in the study; nine of them withdrew, and five returned the questionnaire with missing data. Therefore, the final sample was 260 nurses. Their mean age was 40.61 years (SD = 11.32), with an average of 18.30 years of nursing experience (SD = 9.79). The average mean of their monthly income was 703.08 Jordanian Dinars (SD = 131.14), and the average hospital size was 149.81 beds (SD = 27.93). The sample was predominantly females (71.92%), with males comprising a percentage of 28.08%. Moreover, the majority hold a bachelor's degree in nursing only (82.31%), and a percentage of 17.69% of them had a master's degree. A Percentage of 16.54% and 16.15% were working in medical-surgical and emergency departments, respectively. Regarding nurse-patient ratios, 53.08% worked in units with a ratio of less than 1:5, signifying fairly satisfactory staffing conditions, 37.31% had a ratio of 1:5–1:10, indicating moderate staffing conditions which might pose challenges during high-demand situations. However, about 9.62% had a ratio of more than 1:10, representing significant staffing shortages that could influence the quality of care, and nurses' satisfaction and retention rate (see Table 1).

### 3.2. The Levels of CLB, Flourishing, and ERB Among Nurses

The results showed that the mean total score of perceived CLB of the nurses working in public hospitals was 138.01 (SD = 7.82). This mean equals a percentage of 50.01%, indicating moderate overall participation in CLB. The details of mean scores of the different subscales were as follows: self-awareness (*M* = 18.18, SD = 6.48), advocacy and empowerment subscale (*M* = 18.03, SD = 5.21), decision-making subscale (*M* = 15.84, SD = 3.52), communication subscale (*M* = 18.43, SD = 4.21), quality and safety subscale (*M* = 20.09, SD = 2.42), teamwork subscale (*M* = 17.16, SD = 5.37), and clinical excellence subscale (*M* = 16.96, SD = 6.25) (see Table 2).

The results also showed that only 30 nurses (11.54%) displayed a high level of participation in CLB (See Table 3).

The results showed that the mean of flourishing total score was 31.76 (SD = 5.49), indicating overall positive well-being among the nurses. Specifically, the highest mean score was for the item of feeling competent and capable in important activities (*M* = 4.10, SD = 2.05), and the lowest mean score was for the item of being engaged and interested in daily activities (*M* = 3.75, SD = 2.05) (See Table 4).

Furthermore, the mean total score of ERB was 23.96 (SD = 3.93), indicating moderate participation in ERB among the nurses. The item “making suggestions to help the organization” has the highest mean score of 3.11 (SD = 1.25) (See Table 5).

### 3.3. The Relationship Between Clinical Leadership Behaviors and Both Flourishing and ERB Among Nurses

The results indicated a moderate positive correlation between CLB and flourishing, with a statistically significant Pearson correlation coefficient (*r* = 0.49, *p*=0.047). A significant positive correlation exists between CLB and ERB, with a Pearson correlation coefficient (*r*) of 0.36 (*p*=0.004). These findings indicated that effective CLB are associated with higher nurses' flourishing and participation in ERB (see Table 6).

### 3.4. The Predictors of Flourishing and ERB Among Nurses

The results indicated that the CLB significantly positively affected flourishing (*β* = 0.64, *p* < 0.001, CI 95%, 0.94 to 0.35). Also, the following factors predicted flourishing: Age (*β* = 2.96, *p*=0.004), duration of nursing experience (*β* = 0.35, *p* < 0.001), monthly income (*β* = 3.04, *p*=0.002), and hospital number of beds (*β* = 0.77, *p* < 0.001). In addition, the following factors significantly predicted ERB among the nurses in Jordan: CLB (*β* = 1.40, *p* < 0.001), age (*β* = 3.66, *p*=0.004), duration of nursing experience (*β* = 1.18, *p* < 0.001), monthly income (*β* = 1.47, *t* = 4.67, *p*=0.002) and hospital number of beds (*β* = 0.26, *p*=0.009) (See Table 7).

## 4. Discussion

### 4.1. The Levels of Clinical Leadership Behaviors, Flourishing, and ERB Among Nurses

This study investigated the relationship between CLB and both flourishing and ERB among nurses in Jordan. The results showed moderate participation in CLB (*M* = 138.01, SD = 7.82). This was inconsistent with the perceived performance of CL among nurses in Ireland, which was low overall. However, the researchers of that study asked nurses to complete the questionnaire after finishing a busy shift, which might have led to bias in responses [25]. It was also higher than the level of perceived leadership practices among nurses in Uganda [26]. However, there are studies that have shown higher levels of clinical leadership behaviors based on the clinical environment. For instance, a study revealed that critical care nurses working in private hospitals in Egypt received significantly higher scores on clinical leadership behaviors than those in public hospitals, indicating that organizational support and resources may significantly improve leadership competencies [6]. Furthermore, two studies revealed that nurses' engagement in CLB in Jordan could be linked to professional socialization, interdisciplinary factors [27], leadership style, hospital type, and shift work [28]. However, the researchers might not select a sample representing the whole population, affecting the study's external validity. Furthermore, context and organizational culture are expected to differ between settings and countries, possibly leading to this difference in CLB.

### 4.2. The Relationship Between CLB and Both Flourishing and ERB Among Nurses

The results of the present study showed a moderate positive correlation between CLB and flourishing (*r* = 0.49, *p*=0.047). This result was supported by a recent study, especially in communication and clinical excellence [6]. It was also supported by a study that revealed that perceived leadership among nurses and midwives affected their well-being and job satisfaction [25]. Nevertheless, the researchers did not assess the instrument's validity. As an example, it was pointed out that transformational leadership style could positively influence how subordinate nurses view their work and themselves, which made them feel valued and appreciated (*n* = 655) [14] and improved the motivation and morale of nurses and physicians in the Netherlands [29]. However, the researcher undertook this study during the COVID-19 pandemic and did not classify the nurses based on their job positions. The results of the present study were in line with the findings of a study that revealed that perceived CLB among Irish nurses and midwives were positively related to job satisfaction and perceived performance [25]. In common, positive CLB could influence organizational culture and nurse satisfaction among nurses in Uganda [26]. However, these researchers used a convenient sample and did to differentiate how the relationship differed between the nurses working at five selected hospitals. The present study's findings were supported by a study conducted in the United States. It concluded that leadership could enhance healthy work environments, improve nurses' perception of working conditions and well-being, and signify a healthier work environment [30]. The correlation between CLB and flourishing was not high. This could be attributed to some mediating factors such as personal experience [31] and psychological capital [32]. These factors can make the relationship complex. However, the context of the present study could affect this relationship. Therefore, exploring such mediating factors is recommended in future studies.

The results of the present study revealed a significant positive correlation between CLB and ERB. This result was also supported by a study conducted in China, which showed that the set of practices of integrated High-Performance Work Systems, such as service orientation and leadership, enhanced nurses' job crafting and ERB among nurses. However, it was a single-site descriptive study [33]. Likewise, a positive moderate relationship between ethical leadership and ERB was established (*N* = 302) [3]. Thereby supporting the call for ethical practices at the organizational level. Nevertheless, this study was descriptive and conducted in one hospital only. Another study reported that CLB enhanced staff involvement and commitment and enabled nurses and physicians to perform tasks beyond their job requirements [29]. However, the response rate was only 46.5%. The strength of the relationship between CLB and ERB is moderate. It could be moderated by the effect of the work environment [34]. Other factors that moderate this relationship could differ in each context.

### 4.3. The Predictors of Flourishing and ERB Among Nurses

The present study's findings showed that flourishing predicted participation in ERB among nurses. Similarly, this finding was supported by a study that concluded a positive relationship between role participation and ERB [1, 3, 25], encouraging nurses to make discretionary efforts by assisting their counterparts and even standing up for patient rights [14, 29] and improved the willingness of nurses to perform more [26].

The regression analysis in the present study revealed that age, hospital number of beds, duration of nursing experience, and monthly income significantly predicted flourishing. This was consistent with previous studies findings that concluded income and economic factors could impact the flourishing of the healthcare systems [6, 33, 35]. This indicates the significance of financial stability in decreasing stress and providing safety, which allows nurses to effectively address their professional responsibilities [6]. Experience was also a significant predictor, as reported in previous studies [3, 26].

In relevance to the results of the present study, age played a crucial role in promoting nurse flourishing [3, 26, 33], giving a boost to human thriving as learned employees were more capable of dealing with stress and had a sense of work satisfaction, thus boosting their well-being [3, 26]. Consistent with the results of the present study, a hospital's number of beds was found to have a role in promoting nurse thriving by providing more means and ways of personal and professional growth. However, some differences were noted across studies that underscore the impact of cultural beliefs, socioeconomics, and system issues. It was documented that organizational culture and work environment are influential in determining nurse flourishing [7, 12]. Therefore, the predictors of flourishing established in this study indicate that leadership characteristics and demographic features interact and underscore the significance of fostering positive work environments and economic status to achieve nurse well-being and flourishing.

The results of the present study signified the importance of age, hospital number of beds, duration of nursing experience, and monthly income in predicting ERB. This finding was supported by a study that revealed that income was another predictor since financial resources enable nurses to devote their time and efforts to nursing duties and perform additional roles [6, 32]. Previous studies revealed that experienced and older nurses were well-placed to go beyond the expected job responsibilities, ERB, and perform extra effort that benefits their organization and coworkers. They possessed more extensive knowledge and experience as registered professionals [3, 26]. Lastly, hospital characteristics affect ERB, whereby large hospitals offer possibilities for professional growth and support to assume extra roles, which challenges nurses [25, 30]. This was consistent with the findings of the present study. This study meaningfully helps in understanding of the relationship between clinical leadership, flourishing, and ERB among Jordanian nurses. The findings revealed moderate levels of clinical leadership behaviors among nurses, emphasizing the importance of conducting leadership training programs to improve nurses' well-being, and enhance the best quality of patient care. The study also indicates a positive link between clinical leadership and nurse flourishing, signifying that supportive organizational leadership can improve nurse satisfaction and ERB.

### 4.4. Limitations

Some possible limitations of this study include the following: First, the design is cross-sectional, which restricts the possibility of establishing causality between the observed CLB, flourishing, and ERB. Conducting longitudinal and qualitative research studies is recommended to further understand the causality between the investigated variables. Second, relying on self-assessment may result in response bias, where participants may only give desired answers. Third, the study enrolled a convenient sample from governmental hospitals, which may limit the generalizability of the findings. Enrolling a sample from private hospitals could provide a comprehensive view and improve the generalizability of the findings. Fourth, potential cultural factors that might influence CL perceptions in Jordan could affect the findings. Future studies are recommended to understand these perceptions further. Finally, despite the fact that all steps were employed to achieve a high response rate, the potential for non-response bias remains, as nurses who refused to participate might differ thoroughly in their perceptions from those who participated in the study.

### 4.5. Implications for Nursing Management

The findings of this study are expected to contribute to nursing practice, leadership advancement, and healthcare policies. The moderate levels of perceived CLB, flourishing, and ERB among nurses support the call for increased training. Moreover, the findings imply that nursing administrators and policymakers are encouraged to facilitate ongoing education and emphasize leadership competencies. In addition, it is important to meet the multifaceted needs of nurses within the clinical context. This could ensure a supportive work environment and improve CLB and ERB.

## 5. Conclusions

The findings significantly contribute to understanding the relationship between CLB, flourishing, and ERB among nurses in Jordan. The results revealed that engaging in CLB improves flourishing and ERB among nurses. CLB, age, duration of experience, income, and hospital number of beds are potential predictors of nurse flourishing and ERB. These findings call for improving CLB to further optimize flourishing and ERB among nurses.

## Figures and Tables

**Table 1:** Sociodemographic and work-related characteristics of nurses (*N* = 260).

Variable	Category	*N*	%	*M*	SD
Age (in years)	—	—	—	40.61	11.32

Duration of nursing experience (in years)	—	—	—	18.30	9.79

Monthly income (in JOD)	—	—	—	703.08	131.14

Hospital beds number	—	—	—	149.81	27.93

Gender	Female	187	71.92	—	—
	Male	73	28.08	—	—

Marital status	Married	151	58.1	—	—
	Single	109	41.9	—	—

Level/degree of education	Bachelor's degree in nursing	214	82.31	—	—
	Master's degree in nursing	46	17.69	—	—

Current job position	Staff nurse	229	88.08	—	—
	Charge nurse	31	11.92	—	—

Specialty area	Medical-surgical	43	16.54	—	—
	Emergency	42	16.15	—	—
	Pediatrics	38	14.62	—	—
	ICU	37	14.23	—	—
	Orthopedics	35	13.46	—	—
	Oncology	34	13.08	—	—
	Psychiatry	31	11.92	—	—

Nurse-patient ratio in unit/department	Less than 1:5	138	53.08	—	—
	1:5–1:10	97	37.3	—	—
	More than 1:10	25	9.62	—	—

*Note: M* = mean, *N* = frequency, % = percentage, JOD = Jordanian Dinar.

Abbreviations: ICU = intensive care unit, SD = standard deviation.

**Table 2:** Levels of clinical leadership behaviors among study participants.

Variable	*M*	SD	Score percentage out of 100
Self-awareness subscale (6 items)	18.18	6.48	—
Advocacy and empowerment subscale (6 items)	18.03	5.21	—
Decision-making subscale (7 items)	15.84	3.52	—
Communication subscale (6 items)	18.43	4.21	—
Quality and safety subscale (7 items)	20.09	2.42	—
Teamwork subscale (6 items)	17.16	5.37	—
Clinical excellence subscale (8 items)	16.96	6.25	—
Total score of clinical leadership behaviors	138.01	7.82	50.01%

**Table 3:** Levels of participation in clinical leadership (CL) behaviors among nurses (*N* = 260).

Participation level	Cut point	*N*	%
Low	0%–33.3%	40	15.38
Moderate	33.4%–66.6%	190	73.08
High	66.7%–100%	30	11.54

*Note: N* = frequency, % = percentage.

**Table 4:** The mean score of items of the flourishing scale (*N* = 260).

Domain	Item	*M*	SD
Flourishing	I lead a purposeful and meaningful life.	3.98	1.99
	My social relationships are supportive and rewarding.	3.94	2.01
	I am engaged and interested in my daily activities.	3.75	2.05
	I actively contribute to the happiness and well-being of others.	4.03	2.00
	I am competent in the activities that are important to me.	4.10	2.05
	I am a good person and live a good life.	4.08	1.87
	I am optimistic about my future.	3.96	1.98
	People respect me.	3.92	2.10
	Total score of flourishing	31.76	5.49

*Note: M* = mean.

Abbreviation: SD = standard deviation.

**Table 5:** The mean score of items of the extra-role behavior scales (*N* = 260).

Domain	Item	*M*	SD
Extra-role behavior	This employee looks for ways to make the organization more successful.	2.93	1.65
	This employee acts to protect the organization from potential problems.	3.06	1.42
	This employee makes suggestions to help the organization.	3.11	1.25
	This employee keeps well-informed about where his/her opinion might benefit the organization.	3.03	1.45
	This employee continues to look for new ways to improve the effectiveness of his/her work.	2.92	1.48
	This employee encourages coworkers to try new and more effective ways of doing their job.	2.97	1.39
	This employee speaks favorably of the organization to other employees.	2.98	1.44
	This employee gains knowledge, skills, and abilities to benefit the organization.	2.91	1.39
	Total score of extra role behavior	23.96	3.93

*Note: M* = Mean.

Abbreviation: SD = standard deviation.

**Table 6:** The relationship between clinical leadership behaviors and both flourishing and extra-role behavior among nurses.

Variable		Clinical leadership behaviors total score
Nurse flourishing total score	*r*	0.489^∗^
	*p*	0.047

Extra-role behavior total score	*r*	0.359^∗∗^
	*p*	0.004

^∗^Indicates *p* < 0.05.

^∗∗^Indicates *p* < 0.01.

**Table 7:** Predictors of flourishing and extra-role behavior among nurses (*N* = 260).

Predictors	B	*p* value	*t*-test	*p* value	95.0% CI	
					Lower	Upper
Flourishing						
Constants	−26.236		−1.14	0.255	0.35	2.59
Clinical leadership score	0.24	0.64	4.30	< 0.001	0.94	−0.35
Age	0.16	2.96	2.91	0.004	0.96	4.96
Duration of experience	7.07	0.35	5.96	< 0.001	4.73	9.40
Monthly income	0.16	3.04	3.18	0.002	4.93	−1.16
Hospital number of beds	0.48	0.77	18.95	< 0.001	0.53	−0.43
Extra-role behavior						
Constants	58.294		1.776	0.077	0.96	4.96
Clinical leadership score	0.32	1.40	3.11	< 0.001	0.47	−0.46
Age	0.15	3.66	3.14	0.004	0.96	4.96
Duration of experience	4.27	1.18	6.12	< 0.001	1.56	4.11
Monthly income	1.08	1.47	4.67	0.002	3.03	2.21
Hospital number of beds	0.82	0.26	9.45	0.009	1.31	−0.56

*Note:* B = unstandardized beta, *t* = *t*-test value, *p* = power.

Abbreviation: CI = confidence interval.

## Data Availability

The data that support the findings of this study are available from the corresponding author upon reasonable request.
